# Metal Concentrations in Sediments of the Alinsaog River, Santa Cruz, Zambales, Central Luzon, Philippines

**DOI:** 10.5696/2156-9614-10.27.200914

**Published:** 2020-08-25

**Authors:** Rowena R. Sazon, Veronica P. Migo

**Affiliations:** 1 Department of Biology, College of Arts and Sciences, President Ramon Magsaysay State University, Zambales, Philippines; 2 Department of Chemical Engineering, College of Engineering and Agro-Industrial Technology, University of the Philippines Los Baños, College, Laguna, Philippines

**Keywords:** Zambales, sediments, stream flow, heavy metals, nickel

## Abstract

**Background.:**

Efforts are needed to evaluate heavy metal concentrations in aquatic sediments which serve as repositories and as sources of contamination of other habitats.

**Objectives.:**

The present study assessed temporal changes in the pH, particle size and concentration of metals in sediments of a mining-affected river in Zambales, Philippines.

**Methods.:**

Sediment samples were collected at different periods in four stations along the river using a modified Van Veen grab. The samples were subjected to quartering, air-drying, and sieved through a mesh of 40 mm prior to X-ray fluorescence spectroscopy analysis using Thermo Scientific Niton XL3t to determine metal concentrations. The sediment particle size was analyzed by the sieve method and soil pH by the electrode method.

**Results.:**

Measured metal concentrations in the sediment were as follows: iron (Fe)> calcium (Ca)> chromium (Cr)> nickel (Ni)> manganese (Mn) with averages of 174.6 mg/g, 7.89 mg/g, 6.54 mg/g, 4.82 mg/g, and 2.75 mg/g dry matter (DM), respectively. The mean pH of riverine sediments was generally neutral, except for Station 4. In terms of particle size, silt and clay fractions increased in the upstream station but decreased in the midstream and downstream stations across the sampling periods. The flooding brought by Typhoon *Koppu* resulted in lowered concentrations of Fe, Mn, Cr, and Ni and an upsurge in Ca and potassium levels.

**Discussion.:**

Most of the heavy metals (Fe, Ni, Cr, Mn) exceeded the probable effect level (PEL) for inorganics in sediments, suggesting that the adverse effects of these metals on the environment and aquatic organisms are expected to occur frequently. In comparison with Station 4, which was less affected by siltation, there was a sign of metal enrichment in the area. This indicates that soil erosion and runoff, which might have been triggered by vegetation loss, mineral extraction, and agricultural activities, had a significant impact on the quality of river sediments.

**Conclusions.:**

The findings of the study point to the need for the formulation and implementation of appropriate regulatory measures for the protection and rehabilitation of the heavy metal-loaded river.

**Competing Interests.:**

The authors declare no competing financial interests.

## Introduction

Pollution of aquatic environments is a global concern. Efforts have been carried out to abate this problem. Traditional water quality control and monitoring approaches are insufficient to protect the quality of surface waters and cannot guarantee the absence of contaminants in the aquatic environment.^1^ Contaminated sediments in freshwater and marine systems can be a source of toxic substances and can directly affect the conditions of bottom fauna. In aquatic ecosystems, sediments serve as the sink and source of contaminants due to their variable physical and chemical properties.^2^ It is therefore important to investigate the presence of pollutants in sediments since they are adsorbed by fine-grained particles and materials in suspension.^3^

Heavy metals and persistent organic chemicals need to be assessed and monitored due to their impacts on the environment and human health. Heavy metals are non-degradable and toxic to organisms, even at low concentrations. They can be transported through the atmosphere or via flowing water and tend to end up in the bottom sediments, soil, and underground water.^4^ Once these elements enter the river system, they tend to bind to particulate matter and adsorb to or co-precipitate with carbonates, oxyhydroxides, sulfides, and clay minerals in the sediments.^5^ Sediment quality can therefore serve as an important indicator in the monitoring of aquatic pollution.

Some heavy metals are metabolically required by organisms in trace amounts but are toxic at high concentrations. Elements that are strongly sorbed to the sediment are not available for assimilation by the bottom-dwelling fauna and other aquatic forms. Changes in the physical and chemical conditions of the environment, however, can mobilize the contaminants and release them into the water.^6^ Once in dissolved form, their bioavailability is increased.

Most of the aquatic environments in the Philippines, being archipelagic in nature, are confronted with issues of metal pollution. This can be due to natural processes and anthropogenic activities such as agriculture, smelting operations, and mining.^7^,^8^ The country ranks third in gold reserves, fourth in copper, and fifth in nickel (Ni).^9^ In Central Luzon, Zambales is well known for rich deposits of Ni and chromite (FeCr_2_O_4_). Since mining entails land modifications, it can expose buried materials such as metals from the earth's surface.^10^ They can be carried to downstream ecosystems through runoff, erosion, and sedimentation.

The present study was conducted to assess temporal changes in the metal concentrations of sediments in the Alinsaog River and to compare their levels against the National Oceanic and Atmospheric Administration (NOAA) screening concentration for inorganics. Likewise, it aimed to establish the influence of sediment pH and texture on metal behavior and to determine the correlation among sediment quality variables. The information generated could provide policymakers with insights in the formulation of regulatory and rehabilitation measures for river restoration and could be used as a basis for immediate planning and implementation of sediment quality management strategies such as metal remediation.

## Methods

**Figure 1:**
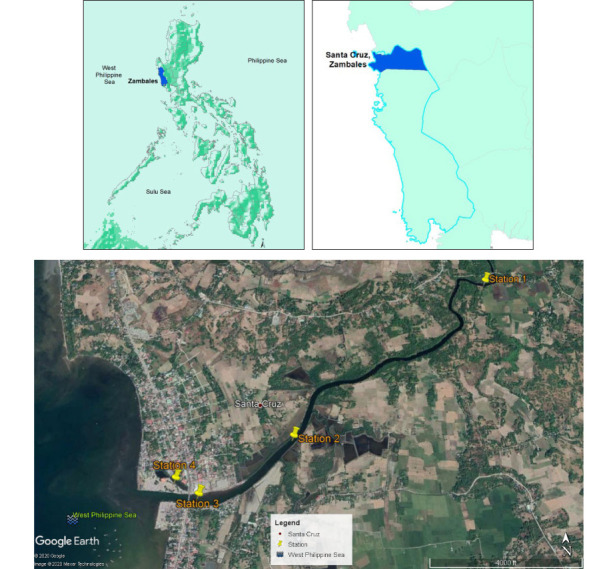
Map showing the location of Santa Cruz and the sampling stations along the Alinsaog River, Zambales, Philippines. Global positioning system coordinates (Station 1: 15.77727 N, 119.93640 E, Station 2: 15.76382 N, 119.91927 E, Station 3: 15.75941 N, 119.91185 E, Station 4: 15.76065 N, 119.90997 E) Satellite image of sampling stations adapted from Sazon et al.^11^

The Alinsaog River, located in Santa Cruz, Zambales, Central Luzon, Philippines, lies at 15°46′1″ N latitude and 119°54′32″ E longitude (*Figure 1*). The river drains its water into the West Philippine Sea. It is a part of the Nayom watershed, where many mining companies operate. The designated sampling stations represent the upstream (Station 1), midstream (Station 2), and downstream (Station 3) sections of the river. Station 4 is a pond-like water body situated close to the river mouth. The riverbanks of Station 1 are dominated by trees and shrubs, whereas patches of *Sonneratia alba*, *Avicennia* sp., *Rhizophora* sp., and *Nypa fruticans* (nipa) thrive at Station 2. *Sonneratia alba* and *Avicennia* sp. prosper at Station 4. Land-based activities in the vicinity of the study area include Ni and chromite mining, aquaculture, and rice farming. Station 3 is surrounded by commercial establishments and fishing communities and serves as a docking site for large fishing boats.

**Abbreviations**
DM: Dry matter
LEL: Low effect level
LOD: Limit of detection
NOAA: National Oceanic and Atmospheric Administration
PEL: Probable effect level
TEL: Threshold effect level
XRF: X-ray fluorescence spectroscopy

### Sediment sampling, preparation and analysis

Using a fabricated Van Veen grab with an area of 2116.2 cm^2^, sampling of the river sediments to a depth of 15–17 cm was performed at the four designated stations (Stations 1–4). Sediments were collected periodically to monitor changes in metal concentrations before the onset of the dry season (October 2014), during the rainy months (August, September 2015), and during the dry season (November 2015).

The three randomly collected sediment samples served as replicates for each station. Prior to the transfer of the sample to a container, water was decanted; however, caution was taken to avoid removing the fine sediment fractions. The composite sediment samples from each station were reduced to a smaller size (250 g) by quartering, air-dried for 3–7 days, pounded using a mortar and pestle, and sieved through a mesh of 40 mm prior to X-ray fluorescence (XRF) spectroscopy determination. The samples were packed in a specific sample container and XRF analysis was done using a Thermo Scientific Niton XL3t in 3 runs at 30 sec/run at the Central Analytical Services Laboratory of the National Institute of Molecular Biology and Biotechnology, University of the Philippines, Los Baños. Another set of composite soil samples from each station was sent to the College of Agriculture Analytical Service Laboratory, University of the Philippines, Los Baños for texture analysis by the sieve method and pH measurement.

Only metals with values above the detection limits were considered, except in cases where one or two of the replications showed a high metal level. If all replications had values below the limit of detection (LOD), the average was reported as <LOD. If one or more replications had <LOD, the LOD value was substituted, but the average was reported as a ≤ value.^12^

### Stream flow measurement

Stream flow or river discharge was measured using the float method described in the Water Quality Monitoring Manual of the Department of Environment and Natural Resources during the months of August and December to represent the wet and dry seasons, respectively.^13^ Two transect lines representing the upstream and downstream sections of each station were laid perpendicular to the river banks. The average depth and width of each transect at four interval points were used to calculate the cross-sectional area of a 6 m long stretch of the river. The time it took for the floating material to travel between transects was determined, and the stream flow was calculated using the following formula.

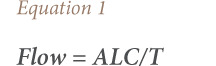
where, A is the average cross-sectional area of a stream, L is the length of the stream reach measured (6 m), C is the coefficient of correction factor (0.8 for rough, rocky bottom; 0.9 for smooth sandy, muddy and smooth bedrock), and T is the time (s) for traveling through L.

### Data analysis

Due to the absence of standard limits for metals in sediments in the Philippines, the quality of the river sediments was evaluated using the NOAA Screening Quick Reference Table for inorganics in sediments.^14^ Metal concentrations were compared against the specified threshold effect level (TEL), low effect level (LEL), and probable effect level (PEL). A factorial analysis of variance was used to determine variations in pH, texture, and metal concentration at the different stations across the periods. A Pearson moment correlation coefficient analysis was likewise performed to determine the association among metals using Statistical Analysis System software.

## Results

*Table 1* presents the mean pH of river sediments across the different stations. The pH ranged from 4.48 to 7.64 and was generally neutral from upstream to downstream (Station 1–3). Station 4, a station with relatively stagnant water and which receives water from Station 3 during high tide, had a slightly acidic mean pH of 4.48 and 4.9 during the wet and dry seasons, respectively.

**Table 1:** pH and Relative Proportions of Sand, Silt and Clay in the Four Stations Across Study Periods

**Station**	**Sampling Period**	**Mean pH**	**Relative Proportion**			**Textural Class**

			**Sand (%)**	**Silt (%)**	**Clay (%)**	
Station 1 (upstream)	Oct. 2014 (A)	ND	95	3	2	sand
	Aug. 2015 (W)	5.26	76	16	8	sandy loam
	Sep. 2015 (W)	7.38	55	24	21	sandy clay loam
	Nov. 2015 (D)	6.44	22	59	19	loam
Station 2 (midstream)	Oct. 2014 (A)	ND	60	26	14	sandy loam
	Aug. 2015 (W)	7.22	76	19	5	loamy sand
	Sep. 2015 (W)	7.21	13	55	32	silty clay loam
	Nov. 2015 (D)	7.36	97	0	3	sand
Station 3 (downstream)	Oct. 2014 (A)	ND	19	35	46	clay
	Aug. 2015 (W)	7.2	57	19	24	sandy clay loam
	Sep. 2015 (W)	7.45	54	27	19	sandy loam
	Nov. 2015 (D)	7.64	63	21	16	sandy loam
Station 4	Oct. 2014 (A)	ND	90	3	7	sand
	Aug. 2015 (W)	7.19	79	13	8	loamy sand
	Sep. 2015 (W)	4.48	60	32	8	sandy loam
	Nov. 2015 (D)	4.90	97	0	3	sand

Abbreviations: A, before the onset of dry season; W, wet season; D, dry season; ND, no data

**Figure 2:**
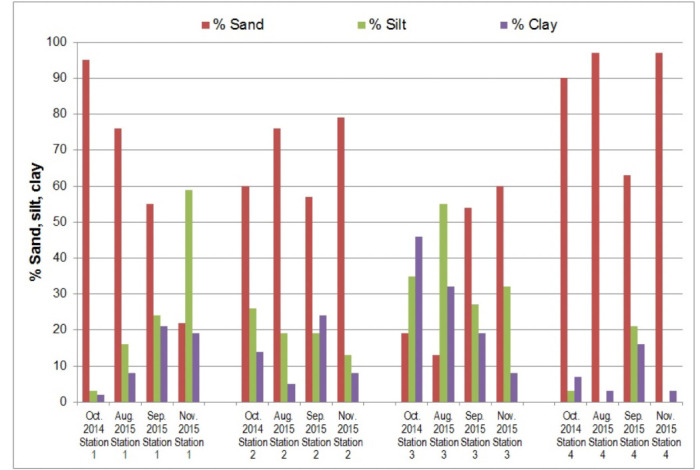
Changes in texture of the sediments in Alinsaog River at different periods (October 2014 to November 2015)

In terms of sediment particle size, the upstream station (Station 1) had increasing silt and clay fractions and decreasing sand fractions across the sampling period (*Table 1, Figure 2*). The texture changed from sand to sandy loam, sandy clay loam and finally to loam across the periods.

In contrast with the upstream station, the downstream station (Station 3) showed an increasing percentage of sand and decreasing fine sediment fraction. The textural grade of the sediments changed from clay to sandy clay loam and finally to sandy loam. The midstream station (Station 2), however, showed a different pattern. The percentage of coarse particles increased while the fine-grained particles decreased from October 2014 to August 2015. The rainy season in September resulted in a reduction in the sand fraction; however, it was followed by an upsurge in sand particles and washing out of the silt and clay fractions after municipality-wide flooding in October of the same year. Station 4, on the other hand, had no observed changes in the soil texture across stations. Station 4 had the highest percentage of sand fractions, ranging from 60–97%.

### Stream flow

The calculated stream flow was found to be lowest upstream (4.5 m^3^/s), followed closely by midstream (4.9 m^3^/s) and the fastest downstream (20.5 m^3^/s) during the dry season (*Table 2*). The same trend was also noted during the wet season, but with relatively higher flow in each station. Among the stations, the stream flow in Station 4 remained generally low, with a value of 0.5 m^3^/s in both seasons.

**Table 2:** Stream Flow (m^3^/s) of Sampling Stations Along the Alinsaog River During the Wet and Dry Seasons (adapted from Sazon et al.^11^)

	**Wet season (August)**				**Dry season (December)**			

	**Station 1 (upstream)**	**Station 2 (midstream)**	**Station 3 (downstream)**	**Station 4**	**Station 1 (upstream)**	**Station 2 (midstream)**	**Station 3 (downstream)**	**Station 4**
Average width (m)	31.0	56.8	105.0	24.0	26.0	52.0	105.0	42.4
Average depth (m)	2.1	1.9	1.1	0.6	1.4	1.1	1.2	0.3
Cross-sectional area (m^2^)	64.9	105.1	115.6	14.7	34.9	57.2	120.8	143
Stream flow (m^3^/s)	11.8	18.7	22.2	0.5	4.5	4.9	20.5	0.5

### Metal concentrations in river sediments

The sediment samples were analyzed by XRF to determine their elemental composition (mg of element per kg of dry sediment). Out of the 28 metals analyzed, only selected metals were shown as the other metals were below the detection limits of the device used.

Among the metals with high recorded concentrations, the sediments had metal levels following the order of iron (Fe)> calcium (Ca)> chromium (Cr)> Ni> manganese (Mn) with averages of 174.6 mg/g, 7.89 mg/g, 6.54 mg/g, 4.82 mg/g, and 2.75 mg/g dry matter (DM), respectively. The measured metal concentrations were compared against the NOAA Screening Level. Table 3 shows that the zinc (Zn) level in the upstream in September (0.123 mg/g) was equal to the threshold effect level (0.123 mg/g) and above the LEL, while the other stations at all period of observations were below the LEL. The Zn levels in Station 4, on the other hand, exceeded the LEL during the rainy months of October and September. Mean concentrations of Ni, Mn, and Cr exceeded the TEL, LEL, and PEL. Iron likewise was above the probable effect concentration level of 40.0 mg/g DM.^15^

**Table 3:** Average Metal Concentrations (mg/g DM) in Sediments of the Alinsaog River Across Four Sampling Periods (October 2014, August 2015, September 2015, November 2015)

**Station**	**Sampling period**	**Metal Concentration (me/g DM)**							

		**Fe**	**Ca**	**Cr**	**Ni**	**Mn**	**Zn**	**V**	**K**
Station 1 (upstream)	Oct. 2014	98.9	5.48	3.70	3.73	2.78	0.046	0.074	0.210
	Aug. 2015	99.4	4.05	2.52	3.29	1.53	0.015	0.067	0.037
	Sep. 2015	257.2	5.96	14.0	7.15	3.93	0.123	0.225	0.210
	Nov. 2015	160.9	7.75	10.1	5.29	2.56	0.083	0.195	0.752
**Average**		**154.1**	**5.81**	**7.58**	**4.87**	**2.70**	**≤ 0.067**	**0.154**	**≤ 0.302**
Station 2 (midstream)	Oct. 2014	137.3	3.01	1.96	4.76	2.70	0.010	< LOD	0.210
	Aug. 2015	133.6	4.90	3.66	3.60	2.34	0.015	< LOD	0.037
	Sep. 2015	299.3	5.10	13.1	7.08	4.18	0.119	0.255	0.210
	Nov. 2015	132.1	16.5	9.01	3.76	2.05	0.071	0.218	0.641
**Average**		**175.6**	**7.38**	**6.93**	**4.80**	**2.82**	**≤ 0.054**	**≤0.131**	**0.275**
Station 3 (downstream)	Oct. 2014	284.6	1.63	1.78	6.31	3.71	0.010	0.056	0.210
	Aug. 2015	196.6	3.01	1.88	4.57	2.52	0.015	< LOD	0.210
	Sep. 2015	213.4	8.77	8.50	5.96	3.02	0.107	0.228	0.210
	Nov. 2015	82.3	28.51	8.27	2.33	1.65	0.069	0.195	0.514
**Average**		**194.2**	**10.48**	**5.11**	**4.79**	**2.73**	**≤ 0.050**	**≤ 0.125**	**≤ 0.286**
**Average (upstream, midstream, downstream)**		**174.6**	**7.89**	**6.54**	**4.82**	**2.75**	**≤ 0.057**	**≤ 0.137**	**0.288**
Station 4	Oct. 2014	69.4	11.9	2.10	1.49	0.84	0.131	< LOD	0.821
	Aug. 2015	64.0	14.1	1.95	1.54	0.83	0.061	0.073	0.532
	Sep. 2015	103.2	18.4	6.81	2.38	1.20	0.144	0.207	1.938
	Nov. 2015	84.4	19.2	3.57	2.02	0.90	0.113	0.151	2.090
**Average**		**80.3**	**15.9**	**3.61**	**1.86**	**0.94**	**0.112**	**≤ 0.115**	**1.35**
Limit of detection (mg/g)		0.040	0.160	0.050	0.050	0.090	0.010	0.030	0.210
NOAA (mg/g DM)		20.0 ^a^		0.037	0.018		0.123		
TEL/TEC									
NOAA (mg/g DM) LEL				0.026	0.016	0.460	0.120		
NOAA (mg/g DM)		40.0 ^b^		0.090	0.036		0.315		
PEL/PEC									

Abbreviations: V, vanadium; K, potassium; TEC, threshold effect concentration (below which harmful effects are unlikely to be observed); PEC, probable effect concentration (above which harmful effects are likely to be observed).^15^

^a^TEC

^b^ PEC^15^

PEL: Level above which adverse effects are likely to occur.

TEL: Level below which adverse effects on bottom dwelling fauna are less likely to occur.

**Figure 3:**
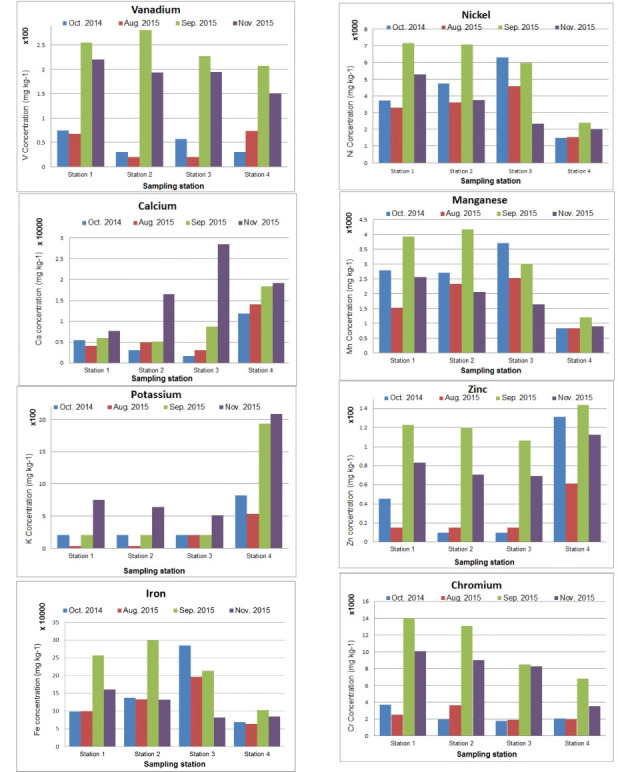
Temporal changes in the concentration (mg/kg DM) of selected metals at the four sampling stations Along Alinsaog River, Santa Cruz, Zambales

*Figure 3* presents the temporal changes in metal concentrations at each station. The heavy metals Fe, Cr, Ni, and Mn had a parallel trend. The highest concentration was noted in September and subsequently decreased in November in most stations except downstream (Station 3). The same trend was observed for Zn and vanadium (V) at all stations. Conversely, the levels of Ca and potassium (K) increased remarkably after flooding at all stations in November sampling, except for Ca at Station 4. The trend for these two metals generally increased from its initial observation in October 2014 to November 2015.

### Sediment quality variables as affected by sampling station and period

*Table 4* presents a summary of the results of the factorial analysis of variance of the sediment quality variables. Other metals detected in the study were included in the analysis. The pH and total elemental concentration of selected metals (strontium (Sr), rubidium (Rb), Ni, Mn, K) had highly significant differences, while Fe, Cr, Ca, and V showed significant differences across stations. There was no significant variation in the particle size of the sediments across stations and periods.

**Table 4:** Summary of Analysis of Variance of Sediment Quality Parameters Across Stations and Periods

**Sources of Variation**		

**Variables**	**Station**	**Period**
pH	0.0008 ^**^	0.6538 ns
% sand	0.6780 ns	0.0957 ns
% silt	0.9120 ns	0.1025 ns
% clay	0.2750 ns	0.1507 ns
Ba	0.3642 ns	0.1823 ns
Sr	0.0006 ^**^	0.0583 ns
Rb	0.0004 ^**^	0.0702 ns
As	0.4217 ns	0.3343 ns
Zn	0.2096 ns	0.2346 ns
Ni	0.0089 ^**^	0.0184 ^**^
Co	0.3133 ns	0.0735 ns
Fe	0.0244 ^*^	0.0366 ^*^
Mn	0.0014 ^**^	0.0163 ^*^
Cr	0.0336 ^*^	0.4255 ns
V	0.0111 ^*^	0.0747 ns
Ti	0.5172 ns	0.1780 ns
Sc	0.6085 ns	0.1950 ns
Ca	0.0353 ^*^	0.0239 ^*^
K	<0.0001 ^**^	0.0375 ^*^

^*^ significant difference at 5% level of significance

^**^ highly significant difference at 1% level of significance

Abbreviations: ns, no significant difference: Ba, barium; Co, cobalt; Ti, titanium; Sc, scandium.

*Table 5* presents the variables whose values differed across the station as revealed by Duncan's multiple range test at a 5% level of significance. The pH of the upstream, middle, and downstream sections of the river (Stations 1–3) were not significantly different from each other but differed from Station 4. For metals whose values showed significant differences in analysis of variance *(Table 4),* further analysis by Duncan's multiple range test revealed that the levels along the river (Stations 1–3) varied from those in Stations 4. Station 4 had relatively higher levels of Sr, Rb, and K, but lower concentrations of Mn, Ni, and V. Iron and Cr levels were higher in the river, however their values in the downstream station were not significantly different from Station 4. The level of Ca at the upstream station was significantly different from that in Station 4, although it did not vary significantly from the mid- and downstream stations.

**Table 5:** Summary of Results of Duncan's Multiple Range Test on Sediment Quality Parameters

**Variables**	**Station 1**	**Station 2**	**Station 3**	**Station 4**
pH	7.0 A	7.4 A	7.5 A	4.9 B
% sand	53.0 A	54.2 A	70.7 A	62.3 A
% silt	28.2 A	24.8 A	20.5 A	25.3 A
% clay	18.8 A	21.0 A	8.8 A	12.3 A
Sr	34.2 B	48.9 B	50.3 B	85.5 A
Rb	4.0 B	4.0 B	4.0 B	7.9 A
Ni	5289.4 A	4895.1 A	3922.5 A	1985.6 B
Fe	198438.0 A	188283.0 A	148594.0 AB	87542.0 B
Mn	2859.3 A	2827.3 A	2031.6 A	991.2 B
Cr	10793.0 A	13005.0 A	9313 AB	4764 B
V	2101 A	236.3 A	216.43 A	165.10 B
Ca	7219.0 B	12858.0 AB	16195 AB	21319 A
K	606.5 B	330.9 B	364.3 B	1903.0 A

Means with the same letter in the same row are not significantly different from each other at a 5% level of significance (Duncan's multiple range test).

Means with the same letter in the same row are not significantly different from each other at a 5% level of significance (Duncan's multiple range test).

Duncan's multiple range tests likewise showed that the concentrations of selected metals such as Ni, Fe, Mn, Ca, and K were significantly different across the sampling period (*Table 4, Table 6*). The sampling in September represented the wet season, while sampling in December took place more than five weeks after flooding. Changes in the concentrations of Ni, Fe, and Mn and those of Ca and K were statistically significant across the seasons.

**Table 6:** Summary of Results of Duncan's Multiple Range Test on Metal Concentrations Before and After Flooding

**Metal**	**Before flooding (September)**	**After flooding (November)**	**Direction of change**
Ni	4839 A	3202. B	Decrease
Fe	183815 A	127613 B	Decrease
Mn	2755 A	1779.9 B	Decrease
Ca	10545 B	18251 A	Increase
K	606 B	996 A	Increase

Means with the same letter in the same row are not significantly different from each other at a 5% level of significance.

### Association of sediment pH, texture, and metal concentrations

The behavior and fate of metals can be influenced by pH and sediment texture. *Table 7* shows that Ni, Mn, Cr, and V had a significant positive correlation, while Sr, Rb, K, and titanium (Ti) had a negative correlation with sediment pH. Pearson correlation coefficients likewise showed a significant negative association between the concentrations of Ni, Fe, cobalt (Co), and Mn and the percentage of sand and a positive correlation with fine sediment fractions. The elements barium (Ba), Ca, and Sr were found to be positively correlated with sand fractions. Among the metals, there was a positive association among Ba, Ca, and Sr and among Ni, Co, Fe, Mn, and V. A negative association also existed between these two metal groups (*data not shown*).

**Table 7:**
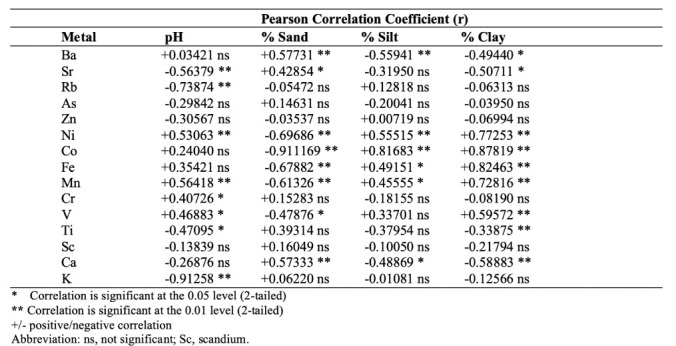
Pearson's Correlation Coefficients (r) of Sediment pH, Texture and Metal Concentrations

## Discussion

The pH and texture are valuable in explaining the behavior of metals in aquatic environments. No standard limit for sediment pH has yet been established in the Philippines, but low pH can influence other attributes of the river. The mean pH of 4.48 and 4.9 at Station 4 in the months of September and November, respectively, indicate acidic conditions. This condition could affect metal mobility and bioavailability. Metals, when trapped in sediments, may become unavailable to living forms. Factors such as pH and redox reaction, however, could mobilize these metals from sediments to the water column. When the pH is lowered, the solubility of most metals increases.^16^ Metal mobility is enhanced at a pH below 5.^17^ Metals tend to desorb from the soil material at lower pH and adsorb or precipitate onto solid materials at higher pH.^18^ Nickel likewise increased its solubility in acidic conditions.^4^ The results suggested that metals that are bound to the sediments in Station 4 have a tendency to desorb from the sediments and be released into the overlying water; hence, metals could exert their toxicity on aquatic organisms. A pH of less than 5.5 is harmful to freshwater shrimp, snails, and clams.^19^

Temporal changes in the texture of the river sediments can be due to differences in stream flow. Stream flow refers to the volumetric discharge or volume of water moving over time in a stream or channel.^20^ Stream flow varies between seasons and among stations. Stream flow was highest during the wet season. Increasing width but decreasing depth was recorded from upstream to downstream (Station 1 to Station 3), which was parallel to the increasing cross-sectional area of each station. Zambales has a Type 1 climate, which is characterized by very pronounced dry and wet seasons. The dry season occurs from November to April, while the remainder of the months are considered the wet season. The meteorological data obtained from the Philippine Atmospheric, Geophysical and Astronomical Services Administration synoptic station in Iba, Zambales showed an increase in precipitation (mm) in the months of September and October (*data not shown*). A high amount of rainfall could bring high volumes of water in the channel. The dry season, on the other hand, is characterized by low precipitation, including low water level and less sediment transport. A study in Panay Island likewise showed that river discharge was higher during the wet season.^21^

The stream flow was fastest downstream and slower at the upstream location. Downstream recorded an increasing percentage of sand and decreasing silt and clay particles. When the stream flow is high, more stress is exerted on the riverbed, which causes greater transport of fine-grained particles.^22^ Except for the upstream station, sand fractions increased while silt and clay decreased in November, a month after flooding (*Figure 2*). A large volume of water from the denuded mountain gushed into the river during the height of typhoon *Koppu* and was accompanied by erosion of the river banks upstream. The high river discharge and abrupt increase in water level during the peak of the typhoon *Koppu* might have transported sediments towards the coast of the West Philippine Sea. There was a lower flow in the upstream (11.8 m^3^/s, 4.5 m^3^/s); hence, finer sediments accumulated in this station. With limited water flow, sediments might remain suspended or settle at the bottom of the channel.^22^ It is apparent that the upstream area originally possessed a sandy-to-rocky substrate, considering the difficulty in sampling by grab encountered in the middle portion of the river channel. Siltation might have contributed to the alteration of river sediment characteristics.

The river discharge in Station 4 was the lowest. This station was connected to the downstream section via a small ditch, which allowed the entry of water during high tide only. Furthermore, the movement of water in this station was limited due to its enclosed feature; hence, it was less affected by sediment transport and changes in water level.

### Metal concentrations in river sediments

The metals of concern at the study sites (Fe> Ca> Cr> Ni> Mn) were similar to those in the Mogpog and Boac River in the Marinduque Islands, except for the presence of lead and cadmium, and in the Mangonbangon River (Fe>Mn>Zn>Cu>Cr>Ni) in Tacloban City, Philippines, both evaluated after 20 years and 30 years of post-mining operations, respectively.^23^,^24^ The Ganga River in India and the Owalla Reservoir in Nigeria likewise showed the same trend (Fe>Mn>Zn>Cr>Cu>Ni), except that Mn showed higher levels than Cr and Ni.^25^,^26^ Furthermore, similar to the present study, Fe was found to have the highest average concentration in the rivers studied by Senoro *et al*., Decena *et al*., Pandey and Singh, and Aduwo & Adeniyi and the Asa River in Ilorin, Nigeria.^23^–^27^ However, the study area still recorded the highest concentration of Fe (299 mg/g). Moreover, the levels of Fe, Cr, Ni, and Zn at the studied sites were far higher than those of Kasardi River in Mumbai, India, and rivers in Marinduque and Peninsular, Malaysia.^23^,^28^,^29^

For most heavy metals, including Fe, Cr, Ni, Mn and Zn as well as V, the highest concentrations were recorded during the rainy season (September), except at the downstream station.

For most heavy metals including Fe, Cr, Ni, Mn, and Zn as well as V, the highest concentrations were recorded during the rainy season (September), except at the downstream station for Fe, Ni, and Mn. A previous study also reported higher concentrations of selected metals (Fe, Cr) upstream during the rainy season.^26^ The high amount of precipitation during this period resulted in run-off and erosion of mountain soil and brought a large volume of water in the channel that transported the metal-loaded sediments. Contaminants that are long buried in river sediments can potentially be resuspended in the water column by storms, high flows, and changes in river discharge.^30^ The upstream station, moreover, was connected to a tributary situated near a mining site. The elevated Fe level was possibly brought about by the Fe- and Ni-rich eroded materials, characteristic of laterites on mafic and ultramafic rocks in the Zambales Ophiolite complex. Laterite is a highly weathered soil that contains a high amount of Fe oxide and is derived from the weathering of rocks under strongly oxidizing and leaching conditions.^31^ Since the open pit method being employed in the extraction of minerals entails the removal of forest vegetation, this could exacerbate erosion and runoff. Moreover, about 8% of the total land area of Zambales is devoted to agricultural production, 60% of which is allotted to rice farming. Bordering the river are farmlands where inorganic fertilizers and pesticides can be potential sources of heavy metal contamination, particularly when the river water overflows during typhoons and heavy rainfall. In the downstream station, the high volume of water passing through the waterway transported the metals in the fine-grained sediments to the sea. These clay and silt particles are more effective in accumulating metals than coarser sand sediment fractions.^32^–^34^

The increasing trend and remarkably high amount of Ca in Station 4 (11.9–19.2 mg/g), although the mean was not significantly different from the downstream and midstream stations, could be due to the presence of dead shelled organisms buried in the sediments. The finer sediments were flushed out during flooding, consequently uncovering the naturally Ca-rich sediments in the area prior to siltation. In a separate study by Sazon, the station recorded a density of 127.8 and 154 individuals/m^2^ of mollusks (family Cerithiidae) during the wet and dry seasons, respectively.^35^ This taxon was also present at the mid- and downstream, but at a relatively low density. The presence of snails (gastropods), particularly at Station 3 and 4, was an indication of high concentration of Ca carbonate in the study area, as this compound is essential for shell construction of the shelled fauna.^36^ Furthermore, Ca could be a product of the natural weathering of rocks that are likely rich in calcite and dolomite. These carbonate mineral groups are common impurities of chromite ore, the major local source of the Zambales ophiolite.^37^,^38^ The upsurge in K at Station 4 (0.821–2.01 mg/g), on the other hand, could be a result of runoff from rice farms that bound the floodplains at the mid- and downstream stations. The rice farming communities in the area highly depend on synthetic, inorganic fertilizers to improve their crop harvests.

There were elevated levels of Fe, Ni, Cr, Mn, and V along the river. Nevertheless, their concentrations were lower at Station 4, which was connected to Station 3 only via a small ditch. This supports the conclusion that enrichment of heavy metals occurs along the river. Although reports show that the study area is geologically rich in Cr and Ni deposits, the findings suggest that mineral extraction and other land-based activities contributed to the enrichment of heavy metals in the area under study.^39^, ^40^ This could possibly be due to the continuous flow and deposition of metal-loaded silt from the tributaries in the watershed cascading down the river. This is of serious concern as succeeding studies showed evidence of heavy metal contamination, particularly Cr and Ni, in the floodplains/agricultural farms adjacent to the Alinsaog River. Strong typhoons have triggered flooding along the river and low-lying floodplains, carrying metal-loaded sediments. In 2018, a rice farm situated close to the river recorded high levels of total available Ni and Cr of 2648–2783 mg/kg and 4314–4520 mg/kg, respectively.^41^ Likewise, the potential health impacts of siltation in crop fields affected by river overflow were evaluated by determining the levels of heavy metals in rice grains in 2018.^42^ The average concentrations of Ni and Cr in the grains were 50.09 mg/kg and 40.47 mg/kg, respectively. This exceeded the permissible limit of the World Health Organization (WHO) for Ni (10 mg/kg) and Cr (1.3 m/kg) in plants.^43^ For a daily intake of <200 μg Cr through foodstuff, <10 μg is absorbed by the human body.^44^ As rice is a staple food in the country, the potential health risks associated with its consumption cannot be underestimated.

### Sediment quality and its ecological and human health implications

Sediment quality guidelines were used to serve as a reference point for evaluating the potential of an element to cause adverse biological effects in aquatic ecosystems.^45^ The concentration of Zn was less than the PEL but exceeded the LEL, which signifies that the river can be considered to be clean-to-marginally polluted in terms of Zn and that Zn presently presents a potential risk to aquatic organisms.^45^ The other stations with low values indicated the rare occurrence of adverse effects of Zn on sediment dwelling fauna. The level of Zn in the study area was lower than that of Pagtaban River in Negros Oriental.^46^ Other heavy metals, including Ni, Cr, and Fe, exceeded the PEL, indicating probable adverse biological impacts on organisms in the surface sediments. For Mn, the recorded values exceeded the LEL (0.46 mg/g DM) indicating that the levels of Mn cannot be tolerated by a majority of the bottom dwelling organism. Iron levels likewise exceeded the probable effect concentration, suggesting that the adverse effects of this metal on aquatic biota and the ecosystem are expected to occur frequently. Iron and Cr were several times higher than the PEL. Iron (64.0–299 mg/g DM) was approximately one and a half- to seven-fold higher, and Cr (1.78–14.0 mg/g DM) was twenty-fold to more than a hundred-fold higher than the PEC/PEL. There were no available threshold limits for V, Ca, and K. Several studies have shown that rivers impacted by mining and industrial activities were loaded with heavy metals that were beyond the permissible limits, such as Cr, Ni and Zn in Negros, and Cr, Fe, Zn, and Ni in Mumbai.^24^,^28^

Metals can adversely affect aquatic species, particularly the benthos, considering that metals can be “locked up” in the bottom sediments for a couple of years.^47^ Sediment-dwelling organisms are exposed to metals through the uptake of interstitial waters, ingestion, and via the food chain.^5^ Iron, Ni, Mn, and Zn are essential nutrients that are required for various physiological functions of organisms. Moreover, Cr, Ni, and Zn are environmentally important heavy metals due to their toxicity.^48^ High concentrations of these toxic heavy metals in riverine sediments may pose risks to bottom dwelling organisms.^24^ Nickel, when available in water, can accumulate in biological organisms such as plankton and other bioindicators of water pollution.^4^

Extremely high concentrations of Ni are a threat to biota in water.^49^ An extensive review was conducted by Mwamburi on the levels of various Cr species that could exhibit toxic effects on benthos, insects, crustaceans, and vertebrates including fishes and aquatic plants.^50^ The ecological impacts of Cr, Fe and other heavy metals have also been reported.^51^ The effects of Fe contamination on fish diversity and abundance of periphyton and benthic invertebrates were described by Vuori.^52^

These heavy metals also pose risks to public health. Fish are known to be highly affected in an aquatic system. The rate of heavy metal storage is faster than their excretion or the rate at which they are metabolized, thus fish can cause health problems in humans once they are consumed.^53^ All of the studied heavy metals, Cr, Fe, Mn, Ni, and Zn are known to cause severe toxicity to fishes by altering their morphology and physiological and biochemical functions in blood and other tissues.^53^

Several studies have shown that heavy metals can accumulate in fish tissues: Fe, Mn, and Ni were found to accumulate in shellfish (*Tympanostomus fuscatus*), while Fe, Zn, Cr, Co, and Ni were detected in the tissues of Tilapia or *Oreochromis mossambicus.*^54^,^55^ The bioaccumulation of metals differs among aquatic species. Chromium accumulates more in freshwater prawn, followed by frogs, freshwater crabs and snails, and least in fish.^56^ Since heavy metals in the sediments can enter the aquatic food chain and bioaccumulate, they can enter the human body through consumption of contaminated fishes and shellfish, posing health risks to local residents who are dependent on these resources as sources of protein.

Metals that are present in the study area can cause various health concerns. Iron, which had the highest average concentration of 299 mg/g in the present study, can cause a rapid increase in respiration and pulse rate, coagulation of blood vessels, and hypertension.^57^ Its mechanisms of toxicity were thoroughly discussed in the paper of Genchi *et al*.^58^ Chromium, on the other hand, is a systemic toxicant that can induce damage to multiple organs.^7^ Chromium (III) and Cr (VI) are stable forms, and the latter is considered highly toxic and carcinogenic to humans.^51^ Furthermore, Ni is also immunotoxic and carcinogenic, and causes a variety of health effects, depending on the dose and length of exposure.^58^ Manganese, although considered an essential element, when present in the human body at a high concentration can interfere with the absorption of dietary Fe leading to Fe-deficiency anemia.^59^ It is likewise considered neurotoxic and is an established cause of Parkinsonism.^60^

The specific effects of metals in the present study on the environment and aquatic life, however, cannot be deduced since there is currently no information on the form in which the metal exists and its bioavailability to aquatic life. Total Cr measurements alone cannot provide information on its actual environmental impacts.^50^ The total content of heavy metals is only indicative of the degree of contamination, not of their mobility.^61^ Metals are present in insoluble form if they are attached to clay or organic matter.^62^ Only the soluble or dissolved form of the metal is available to the biota. The high level of metals in the sediments does not indicate that they would exert toxicity to the benthic fauna since they might not be in an available form. However, current conditions in the study area require immediate intervention, since any change in environmental conditions, such as lowering of pH, could make these metals available for uptake by aquatic organisms.

### Association of sediment quality variables

Although temporal changes in metal concentrations were observed, some localized increase or decrease (irregular pattern) in the concentration of metals under study could also be due to differences in sediment characteristics such as pH and sediment particle size.

There was a positive correlation between sediment pH and metals such as Ni, Mn, Cr, and V. At higher pH, their concentration in sediments increases. Under basic conditions, Ni sorbed to hydroxides of Fe and Mn, whereas lower pH increases the solubility of most metals, thus reducing their concentration in sediments.^4^ Different species of Cr dominate at varying pH ranges. Chromium (III) becomes less mobile at pH below 5 because of its adsorption to clay and mineral oxides, but Cr in this study was measured and expressed as total Cr.^63^ A positive correlation likewise between fine sediment fractions and levels of Ni, Fe, and Mn confirmed the findings of Mohiuddin *et al*.^33^ Many metals can adsorb or co-precipitate with clay minerals.^5^ The observed lack of correlation between Cr concentration and sediment texture could be explained by the report of Tam and Wong, in which the ability of metals to accumulate in clay and silt was no longer significant in highly contaminated areas.^32^ The positive association of Ba and Ca with sand could be a result of their high levels at Station 4, where the textural grade ranged from loam to loamy sand. Furthermore, there were positive correlations among Ba, Ca, and Sr and among Ni, Co, Fe, Mn, and V. A negative correlation that existed between these two metal groups is likely due to their similar chemical properties because the former group consists of alkaline earth metals, while the latter is comprised of heavy metals. Nickel had a positive association with Fe (r=0.925) and Mn (r=0.931), confirming the reports that hydroxide and oxides of Fe and Mn have scavenging actions on trace metals.^64^ The same article showed a strong positive relationship of Fe and Mn with Cr, Zn, Ni, and Co in estuarine sediments.^64^ In the present study, Co was correlated with Fe and Mn (r=0.82, 0.79, respectively), whereas Cr had no significant correlation with Fe but was weakly associated with Mn (r=0.42). The Forum of European Geological Survey Geochemical Atlas reported that Ni had the same distribution map as Cr, while Mn was closely associated with Cr, Ni, V, and other metals.^65^

## Conclusions

The Alinsaog River faces water and sediment quality issues as a result of land-based activities, including chromite and Ni mining, and agriculture. The present study showed that the sediments are loaded with toxic heavy metals, particularly Fe, Cr, Ni, and Mn as well as the alkaline earth metal Ca. The concentrations of these metals exceeded the maximum allowable level for inorganics in sediments, indicating that these may cause potential damage to the ecosystem, bottom dwelling organisms, and some fish species, considering that heavy metals do not degrade by natural processes and tend to accumulate in the tissues of organisms by biomagnification. These metals also pose potential risks to human health through the consumption of fish, which are the primary protein source for the local community. The findings further showed that the river is not a suitable source of water for aquaculture and irrigation of rice and other crops. It is hoped that the findings of this study will prompt local government units and environmental agencies to vigilantly regulate activities upstream of the river that contribute to erosion and transport of metal-loaded sediments, and to devise strategies to reduce the potential risks of these metals to aquatic organisms and human health. The findings also point to the need for implementation of measures to ensure that the situation in the study area is not worsened. Interventions such as the provision of appropriate structures in the containment of silt and in the management of stockpiles should take into consideration the scale of operations and the magnitude of the impacts of extreme weather events.

### Recommendations

Continuous monitoring of the distribution and levels of heavy metals in sediments of the Alinsaog River and three other rivers within the Nayom watershed must be conducted to prevent further accumulation of toxic metals, and to reduce the health risks associated with the consumption of fish and other aquatic resources in the affected sites. Since the specific effects of these metals on aquatic organisms were not examined in the present study, analysis of metal species is recommended to determine which heavy metal poses higher health risks. Their bioavailability, particularly Cr and Ni, should be explored in future studies. Evaluation of metal accumulation in organisms at higher trophic levels, such as shelled fauna and fishes, which are directly consumed by humans, is also recommended. The impacts of toxic metals on other adjacent ecosystems, such as agricultural lands and aquaculture ponds, should be further explored.
